# Pattern Recognition Receptors (PRRs) Expression and Activation in COVID-19 and Long COVID: From SARS-CoV-2 Escape Mechanisms to Emerging PRR-Targeted Immunotherapies

**DOI:** 10.3390/microorganisms13092176

**Published:** 2025-09-17

**Authors:** Luca Maddaloni, Ginevra Bugani, Matteo Fracella, Camilla Bitossi, Alessandra D’Auria, Francesca Aloisi, Abir Azri, Letizia Santinelli, Manel Ben M’Hadheb, Alessandra Pierangeli, Federica Frasca, Carolina Scagnolari

**Affiliations:** 1 Department of Public Health and Infectious Diseases, Sapienza University of Rome, 00185 Rome, Italy; luca.maddaloni@uniroma1.it (L.M.); ginevra.bugani@uniroma1.it (G.B.); letizia.santinelli@uniroma1.it (L.S.); federica.frasca@uniroma1.it (F.F.); 2 Laboratory of Virology, Department of Molecular Medicine, Sapienza University of Rome, 00185 Rome, Italy; matteo.fracella@uniroma1.it (M.F.); camilla.bitossi@uniroma1.it (C.B.); alessandra.dauria@uniroma1.it (A.D.); aloisi.1948781@studenti.uniroma1.it (F.A.); alessandra.pierangeli@uniroma1.it (A.P.); 3 USCR-SAG Unit, Higher Institute of Biotechnology, University of Monastir, Monastir 5000, Tunisia; abirazri2020@gmail.com; 4 Department of Biological Sciences, College of Science, King Faisal University, Al-Ahsa 31982, Saudi Arabia; benmhadhebmanel@yahoo.fr; 5 Istituto Pasteur Italia-Fondazione Cenci Bolognetti, 00161 Rome, Italy

**Keywords:** PRRs, SARS-CoV-2, COVID-19, IFNs, long COVID, TLRs

## Abstract

Severe acute respiratory syndrome coronavirus 2 (SARS-CoV-2) is recognized by pattern recognition receptors (PRRs), which play a vital role in triggering innate immune responses such as the production of type I and III interferons (IFNs). While modest PRR activation helps to defend against SARS-CoV-2, excessive or sustained activation can cause harmful inflammation and contribute to severe Coronavirus Disease 2019 (COVID-19). Altered expression of Toll-like receptors (TLRs), which are among the most important members of the PRR family members, particularly TLRs 2, 3, 4, 7, 8 and 9, has been strongly linked to COVID-19 severity. Furthermore, retinoic acid-inducible gene I (RIG-I) and melanoma differentiation-associated protein 5 (MDA5), collectively known as RLRs (RIG-I-like receptors), act as sensors that detect SARS-CoV-2 RNA. The expression of these receptors, as well as that of different DNA sensors, varies in patients infected with SARS-CoV-2. Changes in PRR expression, particularly that of TLRs, cyclic GMP-AMP synthase (cGAS), and the stimulator of interferon genes (STING), have also been shown to play a role in the development and persistence of long COVID (LC). However, SARS-CoV-2 has evolved strategies to evade PRR recognition and subsequent signaling pathway activation, contributing to the IFN response dysregulation observed in SARS-CoV-2-infected patients. Nevertheless, PRR agonists and antagonists remain promising therapeutic targets for SARS-CoV-2 infection. This review aims to describe the PRRs involved in recognizing SARS-CoV-2, explore their expression during SARS-CoV-2 infection, and examine their role in determining the severity of both COVID-19 and long-term manifestations of the disease. It also describes the strategies developed by SARS-CoV-2 to evade PRR recognition and activation. Moreover, given the considerable interest in modulating PRR activity as a novel immunotherapy approach, this review will provide a description of PRR agonists and antagonists that have been investigated as antiviral strategies against SARS-CoV-2. This review aims to explore the complex interplay between PRRs and SARS-CoV-2 in depth, considering its implications for prognostic biomarkers, targeted therapeutic strategies and the mechanistic understanding of long LC. Additionally, it outlines future perspectives that could help to address knowledge gaps in PRR-mediated responses during SARS-CoV-2 infection.

## 1. Introduction

Low-pathogenicity coronaviruses (HCoV-229E, HCoV-NL63, HCoV-OC43 and HCoV-HKU1) circulate within the human population and typically cause mild respiratory diseases such as the common cold [1,2]. Conversely, severe acute respiratory syndrome coronavirus 2 (SARS-CoV-2) is considered a highly pathogenic coronavirus. It was added to the Coronaviridae family and β-coronavirus (β-CoV) genus in 2020, alongside SARS-CoV-1 and the Middle East respiratory syndrome-related coronavirus (MERS-CoV) [3]. The global pandemic, which began in 2020 and lasted for more than three years, known as ‘Coronavirus Disease 19 (COVID-19)’, was caused by SARS-CoV-2, which resulted in millions of deaths and overwhelmed health systems [4]. Although the widespread use of anti-spike (S) vaccines has helped to control the severity of SARS-CoV-2 infections, new variants of the virus continually emerge. These SARS-CoV-2 variants pose a particular risk to vulnerable individuals, such as the elderly and those with weakened immune systems, as they can lead to severe illness and death [5]. 

It has been demonstrated that the dysregulation of interferon (IFN) responses coupled with a potent cytokine storm are pivotal immunopathogenic mechanisms that can result in severe SARS-CoV-2 infection outcomes [6]. The recognition of SARS-CoV-2 by various pattern recognition receptors (PRRs) in the innate immune system triggers the production and secretion of type I and type III IFNs (IFN-I and IFN-III) [7], the release of inflammatory cytokines, and the promotion of inflammatory cell death [6]. This can result in a cytokine storm and tissue damage, which can lead to the development of acute respiratory distress syndrome [8,9]. It has been shown that genetic variations in PRRs significantly impact the immune response to SARS-CoV-2, favoring the progression and severity of COVID-19 [10,11,12,13,14]. In this scenario, a single-nucleotide polymorphism in the Toll-like receptor 3 (TLR3) gene has been linked to the severity of COVID-19 [15]. Furthermore, X-linked recessive TLR7 deficiency, mostly found in male SARS-CoV-2 patients younger than 60 years, can be considered a genetic etiology of severe COVID-19 pneumonia, due to its ability to impair the production of IFN-I by blood plasmacytoid dendritic cells (pDCs) [13]. Additionally, TLR7 represents a potential therapeutic target in controlling the SARS-CoV-2 infection in the early stages of the disease [13,16]. As regards, immunostimulants such as imiquimod can enhance TLR7 activation, thereby improving antiviral responses [17]. The susceptibility to and severity of outcomes from SARS-CoV-2 infection may be linked to sex-based differences, which could be explained by the location of TLR7 on the X chromosome. In this context, men are almost twice as likely as women to experience severe outcomes, which could enhance immune responses [18]. Furthermore, the human alleles rs10774671-A and rs1131454-A have been associated with reduced levels of the 2′-5′-oligoadenylate synthetase 1 (OAS1) protein, a factor that contributes to the severity of the symptoms of the SARS-CoV-2 [19]. In addition to genetic variations in PRRs, anti-IFN neutralizing autoantibodies that target IFN-α/ω have been associated with reduced expression of IFN-stimulated genes (ISGs) and life-threatening or fatal cases of COVID-19 [20,21,22,23]. Although significant research has advanced our understanding of the characteristics, distribution and functions of PRRs, their specific role in triggering the immune response to SARS-CoV-2 remains unclear. Further investigation into PRR-mediated responses following SARS-CoV-2 infection could lead to the discovery of new treatments for both acute [24,25] and long-term manifestations of the disease, such as long COVID (LC). LC represents a significant global burden, affecting not only in high-income countries but also Africa [26].

In light of these considerations, this review summarizes the dual role of PRRs in initiating and maintaining an anti-SARS-CoV-2 immune response, as well as their contribution to excessive inflammation and the resulting IFN cascade. Additionally, the review seeks to shed light on the strategies employed by SARS-CoV-2 to evade PRRs recognition and activation. Furthermore, it also highlights differences in PRR expression between COVID-19 and LC. The aim is to explore novel therapeutic strategies that target PRRs and modulate their activity also in the context of long-term symptoms following SARS-CoV-2 infection, for which, despite extensive research, there are currently no fully satisfactory treatments [27]. 

We performed a literature review using the international scientific databases PubMed and Google Scholar to identify published articles reporting data on the IFN response and PRRs, both alone and in the context of SARS-CoV-2 infection. The following search terms were used in combination: “SARS-CoV-2”, “COVID-19”, “Coronavirus Disease 19”, “Interferon”, “IFN”, “Pattern Recognition Receptors”, “PRRs”, “Evasion Strategies” and “Long COVID”. These terms were kept broad to include all applicable studies. The search strategy for terms related to SARS-CoV-2 was limited to full-text articles published between 2020, when the pandemic began, and 2025. We checked the reference lists of relevant articles to identify further articles for analysis.

## 2. The Interferon (IFN) Response

Most of the available information on PRRs involved in IFN production following SARS-CoV-2 infection originates from studies on TLRs, RIG-I-like receptors (RLRs) and DNA sensors. A brief description of these groups of PRRs, along with the IFN system and pathways, can be found below.

### 2.1. Pattern Recognition Receptors

**Figure 1:**
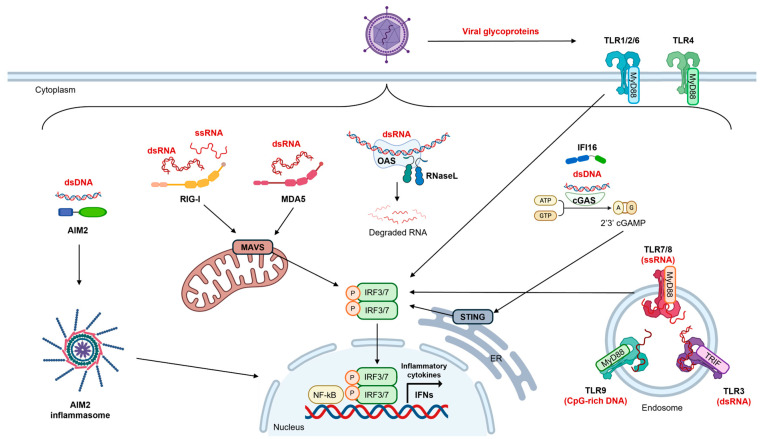
Key viral sensors involved in the induction of interferon (IFN) production during viral infection. The figure illustrates the key viral sensors that are involved in the induction of innate immune response during viral infection, including membrane-bound TLRs and cytoplasmic RNA sensors (such as RIG-I, MDA5 and OAS), as well as DNA sensors (such as cGAS, IFI16 and AIM2). When viral nucleic acids are recognized, these sensors activate signaling cascades that converge on NF-κB and IRF. This results in the transcriptional induction of type I and III interferons (IFNs), pro-inflammatory cytokines and other antiviral effectors. This integrated sensing network is essential for establishing an antiviral state and shaping innate immune responses, as well as orchestrating subsequent adaptive immunity. Phosphorylated proteins are indicated by the letter ‘P’ within an orange-colored circle. Abbreviations: TLRs, Toll-like receptors; RIG-I, retinoic acid-inducible gene I; MDA5, melanoma differentiation-associated protein 5; IFI16, Interferon gamma-inducible protein 16; AIM2, absent in melanoma 2; cGAS, cyclic GMP-AMP synthase; MAVS, mitochondrial antiviral signaling; STING, stimulator of interferon genes; OAS, 2′-5′-oligoadenylate synthetase; MyD88, myeloid differentiation primary response 88; TRIF, TIR-domain-containing adapter-inducing interferon-β; IRF3/7, interferon regulatory factor 3/7; IFN, interferon; NF-kB, nuclear factor-κB; ssRNA, single-strand RNA; dsRNA, double-strand RNA; dsDNA, double-strand DNA; CpG DNA motifs, DNA containing the cytosine-phosphate-guanine dideoxynucleotide motif. Created with BioRender.com.

Following a viral infection, pathogen-associated molecular patterns (PAMPs), such as viral nucleic acids or proteins, and danger-associated molecular patterns (DAMPs), such as high mobility group box 1 (HMGB1), actin, and uridine diphosphate (UDP), are recognized by PRRs, thereby promoting the activation of an early antiviral immune response [28,29,30]. The main PRRs that play a role in inducing IFN-I and III production following viral infection are summarized in Figure 1.

#### 2.1.1. Toll-like Receptors

A prominent group of PRRs involved in IFN induction, through IFN regulated factors 3 and 7 (IRF3/7), are the TLRs [31,32]. Members of the TLR family that have been shown to be involved in responses to viral infection, in part including that of SARS-CoV-2, are TLR1, TLR2, TLR3, TLR4, TLR6, TLR7, TLR8 and TLR9 (Figure 1) [31,32]. TLRs can broadly be divided into two categories: those located at the cell surface (TLR1, TLR2, TLR4 and TLR6) and those in the intracellular endosomal compartment (TLR3, TLR7, TLR8 and TLR9) [32]. It has been demonstrated that TLR2 interacts with a variety of viruses, including both DNA and RNA viruses [32]. Following exposure to a ligand, TLR2 heterodimers activate a myeloid differentiation primary response 88 (MyD88)-dependent signaling pathway which is shared by all TLRs except TLR3 and promotes the nuclear translocation of NF-κB. This pathway stimulates the production of inflammatory cytokines and interleukins, including IFN-I, tumor necrosis factor α (TNF-α), interleukin 1α (IL-1α), IL-1β, IL-6, IL-8 and IL-12 [33,34]. It also activates mitogen-activated protein kinases (MAPKs), which are serine/threonine-specific protein kinases that affect the transcription of inflammatory genes and stabilize mRNA by inducing activating protein-1 (AP-1) [35]. Moreover, TLR2 can induce IFN-I in a specialized class of inflammatory monocytes [32]. In cooperation with TLR2, TLR1 mediates the innate immune response towards lipoproteins of bacterial origin and senses the presence of viruses via their proteins [32]. TLR3 recognizes double-stranded RNA (dsRNA), which is a common intermediate in viral replication, and DNA viruses that generate dsRNA during their life cycle [36]. Following binding to dsRNA, TLR3 activates the TIR-domain-containing adapter-inducing IFN-β (TRIF) signaling pathway, resulting in the production of IFN-β and other inflammatory cytokines [37]. TLR4, the first TLR known to play a role in defending against viruses [38], recognizes a variety of ligands, including lipopolysaccharides, viral glycoproteins (mainly those of RNA viruses) and induces the production of IFN-I [39,40]. It can also sense danger signals produced by necrotic cells and fibrinogen [40]. Although TLR6 is known to be activated by bacterial lipoproteins, recent studies have found that it can also be activated by RNA viruses and may be involved in SARS-CoV-2 infection [41,42,43]. TLR7 and TLR8 are two other TLRs that play a role in IFN production in response to viral infection. They recognize viral single-stranded RNA (ssRNA) and activate the MyD88-dependent signaling pathway, leading to the production of IFN-α [44]. In particular, TLR8 activates MyD88, which then recruits interleukin-1 receptor-associated kinase 4 (IRAK4) to form the Myddosome [45,46]. This complex activates IRAK1, leading to the recruitment of TNF receptor-associated factor 6 (TRAF6) and the lysine 63 (K63)-ubiquitination of IRAK1, resulting in the formation of K63/methionine 1 (M1) ubiquitin hybrids. These hybrids then activate transforming growth factor-β-activated kinase 1 (TAK1) and the IκB kinase (IKK) complexes, thereby triggering the production of pro-inflammatory cytokines via the MAPK and NF-κB pathways [47]. TLR9 binds to unmethylated cytosine-phosphate-guanine (CpG) motifs within DNA molecules. Activation of TLR9 also triggers the MyD88-dependent signaling pathway, leading to the production of IFNs [16,48,49].

#### 2.1.2. Cytoplasmatic RNA Sensors

Another class of PRRs that are involved in IFN induction following viral infection includes retinoic acid-inducible gene I (RIG-I) and melanoma differentiation-associated protein 5 (MDA5). RIG-I binds to short dsRNA (typically less than 1 kilobase in length) and ssRNA with 5′-triphosphate ends. In contrast, MDA5 recognizes long dsRNA typically measuring over 1 kilobase in length. Once viral RNA has been bound, both RIG-I and MDA5 interact with the mitochondrial antiviral signaling protein (MAVS). This triggers a signaling cascade that activates IRF3 and NF-κB. This, in turn, induces the production of IFN and cytokines [50,51,52,53,54].

In addition to RIG-1 and MDA-5, it is also worth noting that the first cytoplasmic proteins discovered to respond to viral dsRNA molecules were the protein kinase RNA-activated (PKR) and OAS proteins (Figure 1) [55]. The OAS family comprises the enzymes OAS1, OAS2, OAS3 and OASL. These enzymes are activated upon binding to viral dsRNAs [56]. They then activate ribonuclease L, which degrades both viral and cellular RNA. This contributes to the induction of an antiviral response and antiproliferative effects [57]. Conversely, PKR trigger a specific translation block after binding to dsRNAs. This impedes viral replication and disrupts the cell cycle [58].

#### 2.1.3. DNA Sensors

In addition to the above PRRs, cytosolic DNA sensors such as cyclic GMP-AMP synthase (cGAS) and absent in melanoma 2 (AIM2) are crucial in triggering the IFN response [59,60]. AIM2 specifically recognizes cytosolic dsDNA from both mammals and viruses. This leads to the recruitment of the apoptosis-associated speck-like protein containing a CARD (ASC) in monocytes and macrophages [61]. This recruitment process forms a complex that activates caspase-1, which results in the maturation of the pro-inflammatory cytokines IL-1β and IL-18 [24,62,63]. In addition, when foreign DNA binds, the cytosolic DNA sensor cGAS catalyzes the production of cyclic GMP-AMP (cGAMP). This acts as a second messenger, activating the stimulator of interferon genes (STING) [50,64,65,66,67,68]. Activation of STING then triggers the downstream signaling pathways involving TANK-binding kinase 1 (TBK1) and IRF3, resulting in the production of IFN [69]. In this context, it has been proposed that STING can detect the fusion of RNA viruses with host cells and may be activated by the IFN-γ-inducible protein 16 (IFI16) inflammasome in response to host DNA damage induced by the virus [70]. The STING/TBK1/IRF pathway then enhances IFN production (Figure 1) and is thought to contribute to Kawasaki-like disease and coagulopathy in COVID-19 [71,72].

### 2.2. The Interferon (IFN) System

In 1957, Isaacs and Lindenmann identified a cellular factor that interfered with influenza virus infection *in vitro* [73]. They named this factor ‘interferon’ (IFN). This finding was significant, setting the stage for further research and leading to a better understanding of the characteristics of IFN. Today, IFNs are a class of antimicrobial, antiproliferative and immunomodulatory proteins produced by most eukaryotic cells in response to various viral inducers and other stimuli. They play a pivotal role in shaping the efficacy of cellular immune responses, by enhancing the presentation of antigens to specific T cells, regulating the activity of B cells, monocytes/macrophages and dendritic cells (DCs), and fostering immune memory [74,75,76]. These activities have long established IFNs as antivirals. They were previously used in combination with ribavirin to treat chronic hepatitis C virus (HCV) infection [77,78], before direct-acting antiviral agents against HCV were developed. IFNs are also used as monotherapy for hepatitis B virus infection and to treat certain cancers and multiple sclerosis [79,80,81,82]. However, sustained or improperly regulated production of IFN during an infection can harm the host organism [83], as also observed in COVID-19 [84]. On the one hand, the occurrence of genetic mutations in the IFN-I pathways or the presence of autoantibodies against IFN-I, which are partly associated with severe cases of SARS-CoV-2 infection, underline the protective role of IFN during SARS-CoV-2 infection; on the other hand, an early elevation in cytokine levels, including the IFN response, which is associated with worse COVID-19 outcomes, highlights its pathogenic role. Consequently, host organisms have evolved sophisticated mechanisms that tightly regulate the timing and specific tissues involved in IFN production [85,86]. These mechanisms also control which pathways and genes are activated in specific cell types as part of the IFN response. 

The IFN family is classified into three types according to sequence, cellular origin, chromosomal location and receptor specificity [86,87]. In humans, IFN-I consists of 13 subtypes of IFN-α, as well as IFN-β, IFN-ω, IFN-ε and IFN-κ in humans (Table 1). While almost all cell types produce IFN-I, the main producers are pDCs (IFN-α), fibroblasts and macrophages [88,89,90]. IFN-II consists of only one component: IFN-γ. This is produced by activated T lymphocytes, natural killer (NK) cells and natural killer T (NKT) cells (Table 1). IFN-γ supports the function of IFN-I and is involved in regulating cell-mediated immune responses. It promotes the activation of macrophages and the presentation of antigens by inducing major histocompatibility complex classes I and II (MHC-I and MHC-II). The last identified class of IFNs, identified approximately 22 years ago, is the IFN-III family, which includes IFN-λ1, IFN-λ2, IFN-λ3 (also known as IL-29, IL-28A and IL-28B, respectively) and IFN-λ4. These are primarily produced by epithelial cells, pDCs, myeloid dendritic cells (mDCs), neutrophils and macrophages (Table 1) [76,91,92,93]. This review focuses primarily on IFN-I and IFN-III because they play a crucial role in the initial innate immune response to viral PAMPs recognized by PRRs. This includes responses triggered by SARS-CoV-2.

**Table 1:** Comparison of human type I, II and III interferons.

IFN Type	Members	Main Cellular Source	Receptor	Receptor Expression	Stimuli	Chromosomal Localization	References
Type I IFN	IFNα subtypes (n = 13), IFNβ, IFNε, IFNκ, IFNω.	pDCs, fibroblasts, macrophages	IFNAR (consisting of two transmembrane domains, IFNAR1 and IFNAR2)	Ubiquitous expression	Viral and microbial components	Chromosome 9	[93,94,95,96,97,98,99]
							
Type II IFN	IFNγ	NK cells, NKT cells, Th1 CD4, Tc CD8	IFNG (consisting of two transmembrane subunits R1 and R2)	Ubiquitous expression	IL-2, IL-12, IL-15, IL-18	Chromosome 12	[50,95,96, 100,101,102,103]
							
Type III IFN	IFNλ1, IFNλ2, IFNλ3, IFNλ4	Epithelial cells, macrophages, pDCs, mDCs, neutrophils	IFNLR (consisting of two subunits, IFNLR1 and IL10Rβ)	Epithelial cells, endothelial cells, macrophages, DCs, neutrophils	Viral and microbial components	Chromosome 19	[95,96,98, 104,105,106]

This table provides a comparative overview of type I, II and III IFNs (IFN-I, II and III), emphasizing their distinct biological roles, signaling pathways and cellular contexts of action. For each type of IFN, information is provided on family members, primary cellular sources, receptors and their expression patterns, typical stimuli and chromosomal localization. Abbreviations: IFN, interferon; pDCs, plasmacytoid dendritic cells; NK cells, natural killer cells; NKT, natural killer T cells; Th1 CD4, T helper CD4^+^ lymphocytes; Tc CD8, T cytotoxic CD8^+^ lymphocytes; mDCs, myeloid dendritic cells; DCs, dendritic cells.

### 2.3. Type I/III Interferon and Signaling Pathways

**Figure 2:**
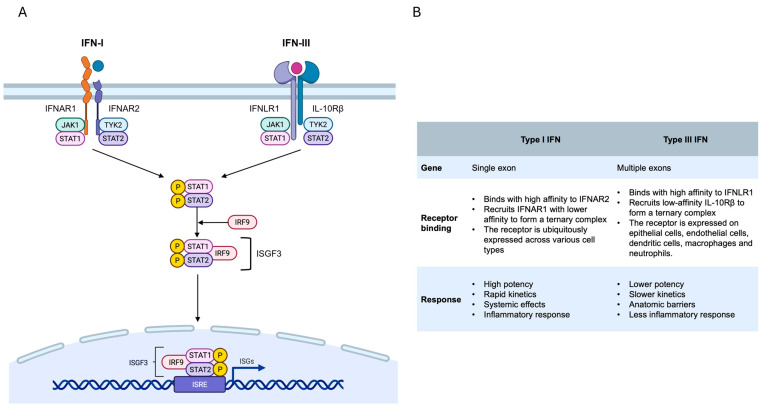
Canonical signaling pathways of type I and type III interferons (IFN-I/III). Panel (**A**): The receptors for IFN-I and IFN-III are heterodimers, consisting of the subunits IFNAR1/IFNAR2 for IFN-I and IL28RA/IL-10R2 for IFN-III. When these receptors dimerize, they activate the kinases TYK2 and JAK1. This activation leads to the phosphorylation of STAT1 and STAT2. Phosphorylated STAT1 and STAT2 then form heterodimers that associate with IRF9 to create the transcription factor ISGF3. ISGF3 then binds to ISRE and promotes the transcription of numerous ISGs. Panel (**B**): Description of the main functional differences between IFN-I and IFN-III, with emphasis on receptor-binding affinities, response specificity and downstream biological effects. While both types of IFN activate overlapping signaling pathways, variations in receptor distribution and binding kinetics result in distinct cellular responses and antiviral activities, reflecting their complementary roles in host defense. Phosphorylated proteins are indicated by the letter ‘P’ within a yellow circle. Abbreviations: IFN-I, type I interferon; IFN-III, type III interferon; IFNAR1/2, interferon-alpha/beta receptor subunit 1/2; TYK2, tyrosine protein kinase 2; JAK1, Janus kinase 1; STAT1/2, signal transducer and activator of transcription 1/2; IRF9, interferon regulatory factor 9; ISGF3, IFN-stimulated gene factor 3; ISGs, interferon-stimulated genes; ISRE, interferon-sensitive response element. Created with BioRender.com.

Although IFN-I and IFN-III are genetically distinct and use different receptors, they are triggered by similar pathogen-sensing pathways and stimulate comparable gene expression programs that promote antiviral, anti-proliferative and immunomodulatory responses [106]. In particular, all IFN-I signals are transmitted via a heterodimeric receptor known as the IFN-alpha/beta receptor (IFNAR), comprising IFNAR1 and IFNAR2 subunits [107]. Conversely, IFN-III transmits signals via a heterodimeric receptor known as IFN lambda receptor (IFNLR). This receptor consists of IFNLR1 [also referred to as IL-28 receptor subunit alpha (IL28Rα)] and IL-10 receptor subunit beta (IL10Rβ) [106]. Despite having different receptors, IFN-I and IFN-III have broadly similar downstream signaling pathways and transcriptional responses. Both types activate the Janus kinase (JAK)/signal transducer and activator of transcription (STAT) pathway, leading to the formation of the heterotrimeric transcription factor complex IFN-stimulated gene factor 3 (ISGF3). This complex consists of phosphorylated STAT1, STAT2 and IFN regulatory factor 9 (IRF9) [50]. Once activated, ISGF3 is transported to the nucleus, where it binds to IFN-stimulated response elements (ISREs). This leads to the transcription of hundreds of ISGs (Figure 2) [7,50,108]. These ISGs target the different stages of the viral life cycle and regulate various cellular processes, including protein synthesis, survival and apoptosis [7,50,109,110,111,112,113]. The different distribution of their receptors is a key factor in the distinct antiviral responses of IFNs. While IFNAR1 and IFNAR2 are found in almost all nucleated cells, the IFNLR complex is located on the surfaces of epithelial cells, endothelial cells, macrophages, DCs, and neutrophils (Table 1) [95,98,106]. As a result, the antiviral properties of IFN-III are most noticeable in the respiratory, gastrointestinal and reproductive tracts [92,105,114]. This response is also associated with the abundance of peroxisomes in epithelial tissues, as these organelles encourage the production of IFN-III rather than IFN-I in the MAVS signaling [115]. Furthermore, although IFN-I/III induce a largely overlapping set of ISGs, their induction kinetics and cell-type specificity differ [106]. The IFN-I response is characterized by its high potency, rapid activation and short-lived nature. This provides an immediate yet transient defense against viral infection. By contrast, the IFN-III response is generally weaker and takes longer to initiate, but provides continuous, prolonged defense. This is particularly effective at maintaining antiviral protection at mucosal and epithelial barriers [116,117,118,119]. This difference may be related to the rapid downregulation of IFN-I signaling by negative regulatory ISGs, such as ubiquitin specific peptidase 18 (USP18), ISG15 and the TAM receptors, Tyro3, Axl and Mer [120,121]. Additionally, IFN-I induces a broad response in different cell types, often resulting in a more potent inflammatory response throughout the body [75]. By contrast, IFN-III induces a more localized response, primarily in epithelial and barrier tissues. This reduces inflammation in these protective areas [106,122,123].

### 2.4. Interferon Stimulated Genes

IFNs exert their cellular functions by regulating the expression of target genes, which are collectively known as ISGs [124]. These genes encode a class of proteins that primarily counteract viral infection and activate immune defenses when activated by IFN-induced pathways [125,126]. To date, almost 300 ISGs have been identified. Each of these genes plays a role in limiting viral replication and spread by performing different antiviral functions. These ISGs cover a wide range of functions, including modulation of intracellular signaling, direct antiviral defense, regulation of inflammation, and adaptation of the immune system [127,128,129]. However, the persistent or impaired production of ISGs and other components of the innate immune response can lead to immunopathology, resulting in damage to virus-infected mucosa [130,131,132].

## 3. SARS-CoV-2 Recognition by Pattern Recognition Receptors

Both the recognition of SARS-CoV-2 and the initiation of the IFN response are greatly influenced by PRRs [9]. Although moderate PRRs activation may offer protection, excessive activation can trigger severe inflammation [133,134,135,136,137] and potentially lead to serious health complications [138,139,140,141], a phenomenon that has also been observed in severe COVID-19 [142]. The primary PRRs involved in the recognition of SARS-CoV-2 are described in the following paragraphs.

### 3.1. SARS-CoV-2 Recognition by Toll-like Receptors

SARS-CoV-2 is sensed by TLRs, which have been identified as critical sensors of this virus [143,144]. In this context, the following TLRs have been associated with the severity of COVID-19: TLR2, TLR3, TLR4, TLR7, TLR8 and TLR9 (Table 2) [145,146].

TLR2 plays a crucial role in the immune response to SARS-CoV-2 by recognizing the viral envelope protein (E) as its ligand in a specific, dose-dependent manner, thereby inducing the release of pro-inflammatory cytokines (Table 2) [36]. Furthermore, the interaction between TLR2 and the SARS-CoV-2 recombinant S protein on NK cells has been shown to have both protective and detrimental effects depending on the stage of infection (Table 2) [147]. In addition to its direct interaction with SARS-CoV-2 components, the activity of TLR2 is functionally linked to host innate immune components that contribute to the immunopathology and severity of COVID-19 (Table 2) [36,148].

Similarly to TLR2, TLR3 appears to play an early role in SARS-CoV-2 infection stimulating the production of various pro-inflammatory cytokines (Table 2) [16]. In terms of its protective function, a deficiency in the TLR3-IRF7-mediated IFN-I response is likely to result in a high mortality rate [149]. However, conflicting reports on TLR3 expression in critically ill patients reflect the complexity of its role as a critical mediator of the immunoprotective and immunopathogenic consequences associated with COVID-19 (Table 2) [150,151]. It is noteworthy that the critical role of TLR3 is emphasized by the possibility that SARS-CoV-2 may employ evasion strategies to avoid the recognition of viral dsRNA by PRRs in a manner similar to SARS-CoV-1 [152]. The role of TLR3 in SARS-CoV-2 infection varies depending on the context and is influenced by the timing of its activation. Timely activation of TLR3 contributes to the initiation and maintenance of effective immune defense, whereas delayed or dysregulated activation can exacerbate inflammation and increase the risk of a cytokine storm, thereby complicating COVID-19 progression [16].

As with TLR2, TLR4 interacts with the SARS-CoV-2 S protein, triggering the production of pro-inflammatory pathways, including IFNs (Table 2) [147,153,154,155,156,157,158]. Importantly, TLR4 plays a dual role in SARS-CoV-2 infection, mediating both protective and harmful effects [159,160]. While blocking TLR4 could reduce hyperinflammation in later stages of SARS-CoV-2 infection, it could also impair IFN-I-mediated immunity in early stages [161,162,163,164,165]. This highlights the importance of timing and context in TLR4-targeted treatments for COVID-19 [166]. *In vivo* evidence of TLR4 impairment has also been reported (Table 2) [167,168,169,170]. As with TLR3, SARS-CoV-2 may hinder TLR4 activation by using the C-type lectin receptor Dendritic Cell-Specific Intercellular adhesion molecule-3-Grabbing Non-integrin (DC-SIGN) as a co-receptor (Table 2) [171]. This could potentially worsen mortality in COVID-19 patients with bacterial superinfection [171].

As previously reported [132,172,173,174], TLR7 activity is essential during SARS-CoV-2 infection, particularly for the induction of IFN-I. Furthermore, several *in vivo* studies have demonstrated an association between SARS-CoV-2 infection and increased TLR7 and TLR8 mRNA expression [175,176]. TLR7 has been shown to play a crucial role in mounting a robust receptor-binding domain (RBD)-specific humoral response to pathogen-like antigens (PLAs)-based AP205-RBD and inactivated SARS-CoV-2 vaccines [177]. Furthermore, it is known that TLR8 recognizes antiphospholipid antibodies, suggesting that it could be used as a prognostic biomarker, particularly for female patients with SARS-CoV-2 infection [178,179].

It is well established that TLR9 is activated by CpG-rich, unmethylated DNA motifs [180]. Although the presence of CpG motifs in the SARS-CoV-2 genome suggests the possibility of direct TLR9 activation, it is unclear whether the virus activates TLR9 via this mechanism [181]. Several hypotheses have been proposed for TLR9 activation during COVID-19, including mitochondrial dysfunction, which is an indirect mechanism related to various health issues and has been observed in different viral infections (Table 2) [180,182,183,184]. TLR9 has also emerged as a promising therapeutic target for preventing and treating COVID-19. The use of TLR9 agonists as vaccine adjuvants has also been suggested [185,186]. Furthermore, elevated levels of TLR9 expression and the presence of TLR9 ligands have been identified as biomarkers for predicting a more severe SARS-CoV-2 infection outcome [181].

Genetic polymorphisms in TLR genes can influence individual susceptibility to, and the outcome of, SARS-CoV-2 infection, highlighting their role in the host immune response (Table 3).

**Table 2:** Toll-like receptors involved in SARS-CoV-2 infection.

**TLRs**	**Immunopathogenesis and Clinical Outcomes**	**References**
TLR2	- The sensing of TLR2 and the SARS-CoV-2 envelope protein (E) occurs prior to viral entry and replication. This results in the release of pro-inflammatory cytokines such as TNF-α, IFN-γ, IL-6, CXCL10, G-CSF, CXCL1, GM-CSF and CCL3.	[36]
	
	- The interaction between TLR2 and the SARS-CoV-2 recombinant S protein triggers intracellular pathways that enhance the activation of natural killer (NK) cells and control SARS-CoV-2 infection in the early stages. However, TLR2 contributes to excessive inflammation in the later stages of SARS-CoV-2 infection.	[147]
	
	- TLR2 activation is linked to the formation of the NLRP3 inflammasome, which exacerbates the inflammatory response during SARS-CoV-2 infection.	[148]
	
	- TLR2 and MyD88 are associated with the severity of COVID-19.	[36]
		
TLR3	- TLR3 induces IFN-α and IFN-β within the first 24 h of SARS-CoV-2 infection via IRF3. This activates the NF-κB pathway, stimulating the production of various pro-inflammatory cytokines, including IL-1α, IL-1β, IL-4 and IL-6.	[16]
	
	- SARS-CoV-2 accessory protein ORF9b hinders the production of IFN-I/III by targeting components of the TLR3-TRIF endosomal RNA-sensing pathway in vitro.	[187]
	
	- Lower levels of TLR3 expression in peripheral blood have been associated with poorer outcomes in patients with severe COVID-19.	[150]
	
	- Neutrophils from patients with severe COVID-19 exhibit higher levels of TLR3 and TLR7 than those from healthy donors. - TLR3 gene expression was found to be significantly higher in critically ill SARS-CoV-2 patients than in those with mild disease.	[151]
		
TLR4	- As observed in murine and human macrophages and NK cells, the interaction between TLR4 and the S1 subunit of the SARS-CoV-2 S protein triggers pro-inflammatory pathways and activates transcription factors such as NF-κB and AP-1. This leads to the production of pro-inflammatory cytokines and IFNs, particularly in pDCs.	[147,153,154,155,156,157,158]
	
	- Higher expression of TLR4 and its downstream signaling mediators, including CD14, MyD88, IRAK1 and TRIF, has been observed in peripheral blood mononuclear cells (PBMCs) from patients with SARS-CoV-2 infection than in healthy individuals. - Increased TLR4 activity has been observed in the myocardium of patients with severe SARS-CoV-2-induced inflammation, which is similar in severity to bacterial sepsis.	[167,168,169,170]
	
	- Downregulation of TLR4 function in human DCs.	[171]
		
TLR7/TLR8	- Peripheral blood pDCs in humans that are deficient in TLR7 produce lower levels of IFN following SARS-CoV-2 infection. - IFN-λ and IFN-γ levels were found to be dysregulated in BAL cells from patients infected with SARS-CoV-2. Patients who died from the infection were found to have lower levels of both TLR7 and TLR8. - Increased interaction was observed between TLR7 and Band 3, a membrane protein found on the surface of mature red blood cells (RBCs), in patients with SARS-CoV-2-associated sepsis. This interaction enhances the ability of red blood cells (RBCs) to bind RNA and act as scavengers. - SARS-CoV-2 infection is associated with increased TLR8 mRNA expression in the nasopharyngeal epithelial cells of patients with COVID-19 compared to healthy individuals. - Patients with severe COVID-19 had significantly higher levels of TLR7 and TLR8 gene expression than those with mild disease.	[132,172,173,174,175,176,178]
		
TLR9	- SARS-CoV-2 induces mitochondrial dysfunction in HUVECs, characterized by increased production of mitochondrial superoxide, altered membrane potential, and increased release of mitochondrial DNA (mtDNA), resulting in TLR9 activation and cytokine secretion.	[180]

TLRs: Toll-like receptors; NLRP3: NOD-like receptor family pyrin domain containing 3; TNF-α: tumor necrosis factor α; IFN-γ: interferon-gamma; IL-6: interleukin-6; CXCL10: C-X-C motif chemokine ligand 10; G-CSF: granulocyte colony-stimulating factor; CXCL1: the chemokine (C-X-C motif) ligand 1; GM-CSF: granulocyte-macrophage colony-stimulating factor; CCL3: chemokine (C-C motif) ligand 3; NK: natural killer; MyD88: myeloid differentiation primary response gene (88); IFN-α: interferon-alpha; IFN-β: interferon-beta; IRF3: interferon regulatory factor 3; NF-κB: nuclear Factor kappa-light-chain-enhancer of activated B cells; IL-1α: interleukin-1 alpha; IL-1β: interleukin-1 beta; IL-4: interleukin-4; TRIF: TIR-domain-containing adaptor-inducing interferon-β; AP-1: Activator Protein-1; pDCs: plasmacytoid dendritic cells; CD14: cluster of differentiation 14; IRAK1: interleukin-1 receptor-associated kinase 1; PBMCs: peripheral blood mononuclear cells; IFN-λ: interferon-lambda; RBCs: red blood cells; HUVECs: human umbilical vein endothelial cells; mtDNA: mitochondrial DNA.

**Table 3:** The association of single-nucleotide polymorphisms in TLR genes with SARS-CoV-2 infection.

TLRs	SNP	Clinical Outcome	References
TLR2	rs5743708	Higher risk of developing pneumonia and severe cases of COVID-19	[188]
			
TLR3	rs3775290	Increased risk of pneumonia in individuals infected with SARS-CoV-2	[189]
			
TLR4	rs4986790	Protective factor in COVID-19	[190]
			
TLR7	rs3853839	Higher severity of COVID-19	[191]
			
TLR9	rs5743836	Susceptibility to and severity of COVID-19	[192]

TLRs: Toll-like receptors; SNP: single-nucleotide polymorphism.

### 3.2. SARS-CoV-2 Recognition by RNA Sensors

SARS-CoV-2-RNA molecules are detected by both RIG-I and MDA5 in the cytoplasm. They play a critical role in the innate immune response by promoting the expression of IFNs and other pro-inflammatory cytokines [193].

#### 3.2.1. RIG-I

One study reported that silencing RIG-I using small interfering RNA (siRNA) in Calu-3 cells significantly decreased the expression of IFN-β expression during SARS-CoV-2 infection [194]. In line with these findings, Change et al. reported that the G protein-coupled receptor ADGRE5 (CD97) acts as a negative regulator of RIG-I by promoting its degradation and interfering with the IFN-I signaling pathway. This consequently facilitates SARS-CoV-2 replication [195]. In contrast, other studies have found that silencing the RIG-I gene does not reduce IFN-β production in Calu-3 cells infected with SARS-CoV-2 [196,197,198]. Nevertheless, deleting RIG-I was found to increase SARS-CoV-2 replication, suggesting that RIG-I plays a role in the antiviral defense system that is independent of the MAVS-IFN signaling pathway [198]. An in vivo study observed that, upon SARS-CoV-2 infection, children with higher basal expression of RIG-I and MDA5 in their upper airway epithelial cells, macrophages, and dendritic cells had stronger innate antiviral responses than adults [199]. Taken together, these findings suggest a potential link between reduced RIG-I-mediated immune responses to SARS-CoV-2, impaired viral clearance, and fatal outcomes [199,200].

#### 3.2.2. MDA5

Another important RLR is the MDA5 protein, which acts as the primary SARS-CoV-2 sensor in human lung cells [196,201]. Studies using both short hairpin RNA-mediated interference and CRISPR-Cas9 knockout techniques have emphasized the crucial role of MDA5 in the detection of SARS-CoV-2 RNA in Calu-3 cells [202]. The MDA5-MAVS-IRF3 pathway has been identified as essential for the induction of IFN-I/III. However, it appears to play only a minor role in the secretion of pro-inflammatory cytokines in response to SARS-CoV-2 infection [202,203,204]. The activity of MDA5 is enhanced by Laboratory of Genetics and Physiology 2 (LGP2), a related helicase that strengthens the IFN response by stabilizing the binding of MDA5 to short dsRNA. Despite lacking the caspase recruitment domain (CARD) necessary for initiating IFN responses, LGP2 enhances MDA5’s sensitivity to viral RNA by facilitating the formation of stable filaments, thereby promoting a stronger and more sustained antiviral state [196,205,206]. Furthermore, IFN production appeared to depend critically on ISG15-mediated ISGylation in the MDA5-mediated antiviral response [207]. Interestingly, the induction of the antiviral protein myxovirus resistance protein A (MxA) mainly occurs in uninfected bystander cells following the recognition of SARS-CoV-2 RNA by MDA5. This highlights the complex regulatory mechanisms involved [202]. Despite the robust production of IFN-I/III and key inflammatory mediators, such as CXCL10, TNF-α and IL-6, in response to the recognition of SARS-CoV-2 by MDA5 in both primary and immortalized lung epithelial cells, this antiviral response alone is insufficient to control SARS-CoV-2 replication [197]. Therefore, the antiviral effects resulting from the recognition of SARS-CoV-2 RNA by MDA5 seem to be limited. Effective control of SARS-CoV-2 replication requires additional immune pathways or external interventions [194,197,208]. A study by Yang et al. showed that the absence of MDA5, RIG-I or MAVS significantly increased SARS-CoV-2 replication in human epithelial cells. Wild-type (WT) cells exhibited an increase in IFN-I and III upon SARS-CoV-2 infection; however, this response was considerably diminished in MDA5-/- and MAVS-/- cells. RIG-I-/- cells maintained moderate IFN signaling; however, their ACE2 expression was found to be around 2.5 times higher than that of WT cells. These results emphasize the vital role of MDA5 in triggering the IFN-I/III response to SARS-CoV-2 and suggest that RIG-I may have an IFN-independent antiviral function [198].

#### 3.2.3. PKR and OAS Family

The dsRNA-dependent PKR and OAS family are integral components of the innate immune response to viral infections. Classified as ISGs, they act as dsRNA sensors [55]. Notably, PKR suppresses translation initiation by phosphorylating eukaryotic initiation factor 2 (eIF2) [209], while also acting as a signal transducer for pro-inflammatory gene expression [210]. The human OAS family consists of four IFN-regulated genes: OAS1, OAS2, OAS3 and OASL. The OAS1-3 enzymes catalyze the production of 2′-5′-linked oligoadenylates, and OASL is known for its synthase activity. These molecules activate RNase L, an endoribonuclease that breaks down single-stranded mRNA and rRNA. This process inhibits protein synthesis, thereby establishing an antiviral state [211]. The direct activation of PKR and OASL by dsRNA has been observed in respiratory epithelial cells and cardiomyocytes infected with SARS-CoV-2. Notably, a connection has been established between the OAS gene family and cardiac injury and failure in patients with severe SARS-CoV-2 infections [212]. Furthermore, recessive single-gene inborn errors in the OAS–RNase L pathway can lead to the uncontrolled production of inflammatory cytokines by mononuclear phagocytes in response to SARS-CoV-2 infection. This could contribute to the development of multisystem inflammatory syndrome (MIS-C) in children [213]. Similarly, monocytic cell lines and primary myeloid cells that are deficient in OAS1, OAS2 or RNase L produce excessive levels of inflammatory cytokines when stimulated by dsRNA or SARS-CoV-2 [213]. Notably, SARS-CoV-2 activates the PKR-mediated integrated stress response (ISR) yet subsequently prevents the formation of stress granules and the expression of ATF4/CHOP [214,215]. Differences were observed between SARS-CoV-2 variants: Delta showed weaker PKR activation, whereas Omicron BA.1 exhibited increased phosphorylation of eIF2α and stress granule formation [215]. More recently, it has been demonstrated that defective RNA processing leads to impaired PKR-mediated antiviral control in brainstem neurons. Specifically, the accumulation of RNA lariats in DBR1-deficient cells disrupts stress granule formation and PKR activation mediated by G3BP. This increases susceptibility to viral infection in both *in vitro* and *in vivo* models of brainstem viral infection, including SARS-CoV-2 [216,217].

### 3.3. SARS-CoV-2 Recognition by Absent in Melanoma 2-like Receptors (ALRs)

ALRs are intracellular sensors of the innate immune system that are mainly induced by IFN-I, but also by IFN-II and other pro-inflammatory cytokines. They belong to the PYHIN family, alongside proteins such as AIM2 and IFI16 [218].

#### 3.3.1. IFI16/p204

IFI16, also known as p204, acts as a sensor of dsDNA viruses. It amplifies antiviral responses by inducing the transcription of RIG-I and promoting the production of IFNs and other antiviral cytokines. It has recently been demonstrated that IFI16 can also sense negative-sense RNA viruses, such as the influenza virus [219]. However, the direct interaction between IFI16 and SARS-CoV-2 has not yet been elucidated. A study by Hamldar et al. found that IFI16 expression levels were significantly higher in people with confirmed SARS-CoV-2 infections than in healthy individuals. A positive correlation was also observed between IFI16 expression levels and symptoms such as skeletal pain [220]. Furthermore, the upregulation of the IFI16 gene in association with IRAK4, STING, IFNAR1 and CD14 was observed in blood cells from patients with moderate-to-severe acute SARS-CoV-2 infections [221]. In this context, IFI16 could be used as a biomarker to distinguish between healthy individuals and those in the acute or post-acute phases of the COVID-19 [220].

#### 3.3.2. AIM2

Similarly to IFI16, AIM2 is a cytoplasmic sensor which recognizes the presence of dsDNA and forms an inflammasome complex known as the AIM2 inflammasome [59,222]. This complex was found to be activated in monocytes isolated from patients with COVID-19 [223]. SARS-CoV-2 genome was found in approximately 6% of blood monocytes of COVID-19 patients. Despite the infection being aborted, these cells undergo pyroptosis, which is mediated by the activation of NLRP3 and AIM2 inflammasomes, as well as by caspase-1 and gasdermin D. Furthermore, tissue-resident macrophages obtained from the lungs of patients who had undergone autopsy and had been confirmed to have SARS-CoV-2 infection have revealed the presence of activated inflammasome [223]. Consistent with this, previous studies have reported elevated levels of IL-1 cytokines in the plasma of COVID-19 patients, as well as evidence of the virus entering myeloid cells *in vitro* and activating the NLRP3 inflammasome and caspase-1 in blood cells [224,225,226].

### 3.4. cGAS-STING Pathway

The cGAS–STING signaling pathway has been identified as a key mediator of inflammation in several contexts, such as infection, cellular stress, tissue damage and autoimmune diseases [227]. While this pathway is primarily activated by cytosolic DNA, there is evidence to suggest that SARS-CoV-2 can activate the cGAS–STING signaling axis [228,229]. STING agonists have consistently been shown to inhibit SARS-CoV-2 infection by inducing IFN-I responses [230,231]. However, high and sustained levels of IFN-I can contribute to immunopathology during the later stages of SARS-CoV-2 infection, leading to heightened inflammation in patients and mouse models [232,233,234,235]. Moreover, Queiroz et al. found that severe cases of COVID-19 were characterized by increased expression of STING and cGAS, as well as elevated plasma levels of IFN-α, IL-6 and TNF-α, compared to non-severe cases. These factors can lead to thromboembolic events and multiple organ failure [233,235,236]. Activation of the cGAS-STING pathway in SARS-CoV-2-infected epithelial cells drives cytokine production via the NF-κB pathway. This highlights the pathway’s role in cytokine responses associated with SARS-CoV-2 infection [232,237]. Studies have shown that the SARS-CoV-2 S protein promotes cell fusion and activates the cGAS–STING pathway by leaking chromatin DNA. This is evidenced by the colocalisation of cGAS with cytosolic genomic DNA in SARS-CoV-2-induced syncytia. Consequently, host self-DNA, including chromosomal and mitochondrial DNA, acts as a danger signal, triggering an IFN-mediated antiviral response [238]. Furthermore, recent studies have revealed that endothelial cells and macrophages play a pivotal role in the dysregulation of cGAS-STING responses [239]. In endothelial cells, mitochondrial dysfunction activates cGAS, leading to the expression of IFN-I and triggering cell activation and death. In macrophages, cGAS activation is triggered by DNA from phagocytosed, dying endothelial cells, primarily inducing IFN-I production [232]. In line with these findings, studies using organ-on-a-chip technology have revealed the presence of SARS-CoV-2 elements in endothelial cells. These elements have been found to be associated with mitochondrial dysfunction and the activation of the cGAS-STING signaling pathway [232].

## 4. SARS-CoV-2 Evasion Strategies by PRRs

**Figure 3:**
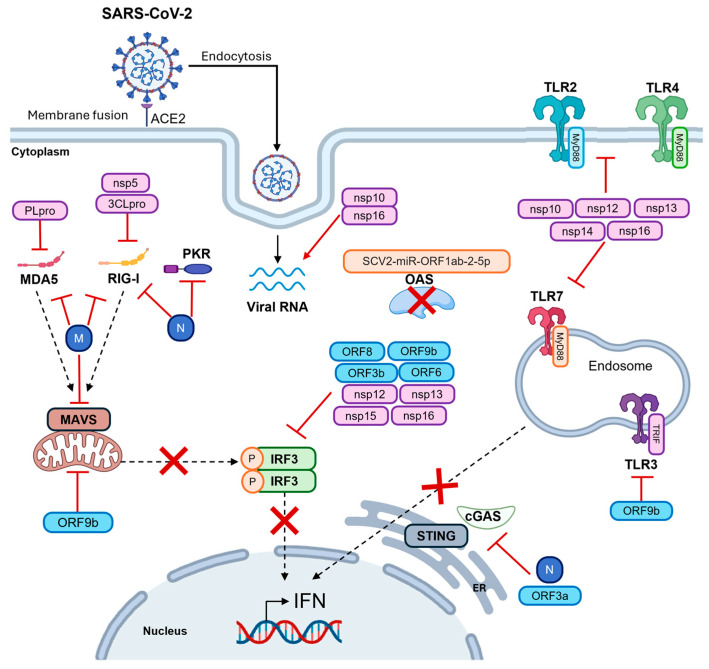
Strategies used by SARS-CoV-2 to evade recognition and signaling by PRRs. The figure illustrates several mechanisms that SARS-CoV-2 employs to evade immune detection by PRRs. The virus modifies its RNA, including 5′ capping by the nsp16/nsp10 complex, as well as 12/13/14/15, in order to mimic host mRNA and evade detection by RNA sensors such as RIG-I and MDA5. Viral proteins, including M, N, 3CLpro and PLpro among others, interfere with key signaling molecules such as MAVS, STING and IRF3, thereby preventing the activation of IFN responses. Furthermore, accessory proteins (ORF3a/b, ORF6, ORF8, ORF9b) and viral microRNAs suppress IFN signaling pathways, collectively dampening the host’s antiviral response and promoting viral replication and pathogenesis. Red crosses indicate sites where the expression of the corresponding host innate immune factor is inhibited. Phosphorylated proteins are indicated by the letter ‘P’ inside an orange circle. Abbreviations: TLRs, Toll-like receptors; RIG-I, retinoic acid-inducible gene I; MDA5, melanoma differentiation-associated protein 5; MAVS, mitochondrial antiviral signaling; cGAS, Cyclic GMP-AMP synthase; STING, stimulator of interferon genes; OAS, 2′-5′-oligoadenylate synthetase; MyD88, myeloid differentiation primary response 88; TRIF, TIR-domain-containing adapter-inducing interferon-β; IRF3, interferon regulatory factor 3; IFN, interferon; NSP, non-structural protein; ORF, open reading frame; 3CLpro, 3C-like protease; PLpro, papain-like protease; M, membrane protein; N, nucleocapsid protein. Created with BioRender.com.

SARS-CoV-2 has evolved multiple mechanisms to evade immune recognition by PRRs at various stages. This dampens the host’s IFN-mediated antiviral response, promoting replication and pathogenesis [240]. The suppression of innate immune signaling pathways also results in the weak and delayed IFN responses observed in COVID-19 patients [9,240]. The main immune evasion strategies of SARS-CoV-2 are summarized in Figure 3. 

One key strategy employed by SARS-CoV-2 is to modify its RNA structure in order to evade recognition by RNA sensors, such as RIG-I and MDA5 [241]. SARS-CoV-2, for example, uses the nsp16/nsp10 heterodimer to methylate the 5′ end of its mRNA. This mimics host mRNA, enabling the virus to evade PRRs detection and hijack the cellular translation machinery [242]. In addition, SARS-CoV-2 encodes its own capping machinery, consisting of nsp10, nsp12, nsp13, nsp14 and nsp16. This machinery further modifies the viral genome, helping SARS-CoV-2 to evade recognition by RLRs and TLRs (including TLR2, TLR3, TLR4 and TLR7) [144,243]. Accordingly, the endoribonuclease activity of nsp15 delays the activation of antiviral responses in human lung cells, making it an important key virulence factor for SARS-CoV-2 [244]. Additionally, the SARS-CoV-2 M protein binds to RIG-I, MAVS and TBK1. This prevents the formation of the RIG-I–MAVS–TRAF3–TBK1 complex, thereby hindering the activation of IRF3 and the subsequent IFN response [245]. The SARS-CoV-2 nucleocapsid (N) protein has been shown to bind the DExD/H domain of RIG-I, thereby interfering with its ATPase activity, which is essential for the recognition of viral RNA [246]. Notably, two mutations in the N protein, R203M (arginine to methionine at position 203) and D377Y (aspartic acid to tyrosine at position 377), have been reported to enhance SARS-CoV-2 infectivity by strengthening the inhibitory effect of the N protein on RIG-I-mediated antiviral signaling [246]. Moreover, SARS-CoV-2 N is endowed with the ability to inhibit PKR activation [247]. Unexpectedly, the N protein of SARS-CoV-2 can bind tightly to DNA, thereby competing with and inhibiting the activation of cGAS [248]. Conversely, the SARS-CoV-2 3C-like protease (3CLpro) prevents the activation of RIG-I by preventing tripartite motif-containing protein 25 (TRIM25)-mediated K63-linked ubiquitination [249], while SARS-CoV-2 papain-like protease (PLpro) antagonizes the ISG15-dependent activation of MDA5 [207]. Furthermore, the SARS-CoV-2 protein ORF9b inhibits the activation of MAVS by preventing the interaction between the translocase of the outer mitochondrial membrane 70 (TOM70) protein and the heat shock protein 90 (HSP90) protein [250]. On the other hand, the Nsp5 protein impairs RIG-I signaling by cleaving its N-terminus, thereby promoting the K48-linked ubiquitination and the subsequent degradation of MAVS [251]. Additionally, the SARS-CoV-2 ORF3a, ORF9b and 3CL proteins inhibit cGAS-STING pathway thereby promoting viral replication [187,229].

High expression of the SARS-CoV-2-encoded microRNA SCV2-miR-ORF1ab-2-5p inhibits the expression of the OAS1 and OAS2 genes, as well as modulating the allelic expression of OAS1. This is associated with high susceptibility to SARS-CoV-2 infection [252]. It has finally been found that structural proteins (N, M) [247,253], and non-structural proteins (PLpro, 3CLpro, nsp12, nsp13, nsp15, nsp16) [254,255,256,257,258,259], as well as accessory proteins (ORF3b, ORF6, ORF8 and ORF9b) [187,260,261,262] contribute to the inhibition of IRF3 activation and its nuclear translocation. This results in the suppression of the IFN response.

## 5. PRRs Agonists and Antagonists in SARS-CoV-2 Infection

Due to their critical role in innate immunity, PRRs have become a focal point of study in immunology and drug development. The variety of PRRs and the broad range of ligands they recognize make them promising therapeutic targets for diseases such as cancer, inflammation, autoimmune disorders and infections caused by pathogenic microorganisms [263]. Their versatility is crucial in developing innovative immunotherapeutic strategies to combat SARS-CoV-2 infection (Table 4).

### 5.1. TLRs Agonists and Antagonists

TLR-targeted immunotherapy can inhibit viral infection, reduce inflammation and enhance the effectiveness of vaccines against SARS-CoV-2 [264,265]. In this context, conjugating the TLR1/2 agonist Pam3CSK4 with the receptor-binding domain (RBD) in a candidate vaccine significantly enhanced antibody and cellular responses. Indeed, sera from immunized mice blocked RBD-ACE2 binding and provided protection against SARS-CoV-2 alpha, beta, gamma and delta variants [266]. Overexpression of TLR1/2 may exacerbate inflammation during SARS-CoV-2 infection. The TLR2 inhibitor oxPAPC has been shown to reduce both cytokine release and mortality in mice that express ACE2. This suggests that TLR2 antagonists could be an effective treatment for severe inflammation in patients with severe COVID-19 [36].

Administering synthetic dsRNA, which mimics viral nucleic acids and activates TLR3 (poly I:C), to K18-hACE2 transgenic mice during SARS-CoV-2 infection improves survival rates by reducing viral load and inflammation in lung and brain tissue [267,268]. Interestingly, the SARS-CoV-2 S protein exhibits the strongest binding affinity to TLR4 [269].

As discussed previously, the regulation of TLR4 may have a dual effect, depending on the stage of SARS-CoV-2 infection at which this modulation occurs [166]. Consequently, targeting this receptor therapeutically could be a way to improve outcomes in severe cases of COVID-19. Resatorvid (also known as CLI-095 or TAK-242) is a TLR4 inhibitor that blocks the interaction between TLR4 and the proteins TIRAP and TRAM. This suppresses signaling in the TLR4/MyD88/NF-κB pathway and the activation of the NLRP3 inflammasome [270]. Conversely, stimulating PBMCs from severe COVID-19 patients, characterized by rare loss-of-function (LOF) variants of the TLR7 gene, with the TLR7 agonist imiquimod (IMQ), revealed an impaired IFN-I response. This was characterized by low levels of IRF7, IFNβ1, ISG15 and IFNγ, highlighting the importance of intact TLR7 signaling in the pathogenesis of severe COVID-19 [144]. In addition, Enpatoran, a selective inhibitor of TLR7/8, can potentially target the pro-inflammatory pathways induced by SARS-CoV-2 infection and reduce the uptake of SARS-CoV-2 RNA by RBCs [173,271]. Finally, the TLR9 agonist CpG-2722 boosts the immune response to the SARS-CoV-2 vaccine by inducing antigen-dependent T helper 1 (Th1) and Th17 responses (Table 4) [186].

### 5.2. RLRs Agonists and Antagonists

The modulation of RLR activity by agonists and antagonists is of considerable interest, given that these molecules have the potential to either enhance antiviral immunity or reduce excessive inflammation, particularly in the context of SARS-CoV-2 infection (Table 4) [272]. Marx et al. demonstrated that treating a K18-hACE2 mouse model of SARS-CoV-2 infection with a RIG-1 agonist triphosphate RNA (3pRNA) protected the mice from lethal infection and promoted the development of specific neutralizing antibodies [273]. In addition, a study showed that the minimal RIG-I agonist stem-loop RNA 14 (SLR14) exhibits antiviral properties, by preventing SARS-CoV-2 infection of the lower respiratory tract and progression to severe disease through an IFN-I-dependent mechanism [274]. Furthermore, given the critical role of MDA5 in triggering antiviral immune responses within the airway epithelium [196], its agonists, such as synthetic poly(I:C) dsRNA and long viral dsRNA [275], have therapeutic potential in combating early-stage SARS-CoV-2 infection (Table 4) [198,276].

### 5.3. Nucleotidyltransferase Family Agonists and Antagonists

The excessive production of inflammatory cytokines, such as IL-6, may be driven by the upregulation of NF-κB via the cGAS-STING pathway. Treatment with the STING inhibitors H-151 and VS-X4 has been shown to effectively reduce levels of TNF and IL-6 in SARS-CoV-2-infected cells *in vitro* [239]. In contrast, the pharmacological activation of the cGAS–STING signaling pathway using the STING agonist diABZI has been shown to inhibit SARS-CoV-2 replication by stimulating the production of ISGs in transgenic mice that have been pretreated with an intranasal treatment and express human ACE2. This treatment has been shown to reduce lung inflammation and increase survival rates [230,231]. Qi et al. demonstrated that glycyrrhetinic acid (GA) mitigated SARS-CoV-2 Omicron infection in Calu-3 and MEF cells, and in mice, by binding to STING and enhancing its phosphorylation. This resulted in elevated levels of CXCL10, IFN-β, OAS1, and ISG15 mRNA [277]. These observations highlight the dual role of cGAS-STING signaling in SARS-CoV-2 infection and pathogenesis, where its hyperactivation contributes to excessive inflammation (Table 4).

**Table 4:** Modulation of PRRs in SARS-CoV-2 infection: effects and therapeutic potential.

Compound	Targeted PRR	Effect of the PRR Modulation	Reference
Pam3CSK4	TLR1/2	Booster of anti-RBD antibody and cellular responses in immunized mice	[266]
			
oxPAPC	TLR2	Reduction in cytokine and chemokine release in ACE2-expressing mice, lowering mortality compared to controls	[36]
			
poly IC	TLR3	Its administration to K18-hACE2 transgenic mice during SARS-CoV-2 infection improves survival by reducing viral load and inflammation in both lung and brain tissue	[267]
			
Resatorvid	TLR4	Suppression of TLR4/MyD88/NF-κB signaling and inhibition of NLRP3 inflammasome activation	[270]
			
IMQ	TLR7	IMQ stimulation on PBMC from severe COVID-19 patients with rare LOF TLR7 variant demonstrated an insufficient induction of IRF7, IFNβ1, and ISG15, as well as a reduction in IFNγ production	[144]
			
Enpatoran	TLR7/8	Enpatoran can reduce the uptake of SARS-CoV-2 RNA by RBCs	[173]
			
CpG-2722	TLR9	Booster of the immune response to SARS-CoV-2 vaccine	[186]
			
3pRNA	RIG-1	Improvement of survival, as evidenced by reduced viral loads in oropharyngeal swabs, lungs and brains of treated mice.	[273]
			
SLR14	RIG-1	Prevention of lower respiratory tract infections and severe COVID-19 disease progression through a type I IFN-dependent mechanism.	[274]
			
H-151 and VS-X4	cGAS-STING	Inhibits STING reducing the level of TNF and IL-6 expression in SARS-CoV-2 infected cells in vitro	[239]
			
diABZI	cGAS–STING	Suppression of SARS-CoV-2 replication by stimulating ISGs production in transgenic mice expressing human ACE2, with a reduced lung inflammation and increased survival rates.	[231]
			
GA	STING	Ameliorated SARS-CoV-2 Omicron infection both inCalu-3 and in MEF cells and in mice. The transcription levels of *Cxcl10*, *Ifnβ*, *Oas1*, and *Isg15* mRNA levels in the MEF cells were up regulated.	[277]

For each listed PRR agonist and antagonist, the specific PRR targets are described, along with their effects on PRR modulation during SARS-CoV-2 infection, including activation or inhibition of downstream signaling pathways. Abbreviations: Pam3CSK4, palmitoyl-3-cysteine-serine-lysine-4; oxPAPC, oxidized 1-palmitoyl-2-arachidonoyl-sn-glycero-3-phosphorylcholine; poly IC, polyinosinic-polycytidylic acid; IMQ, imiquimod; PBMC, peripheral blood mononuclear cells; 3pRNA, triphosphorylated RNA; SLR14, stem-loop RNA 14; diABZI, Di-cyclic amidobenzimidazole; GA, glycyrrhetinic acid.

## 6. Modulation of PRRs in Long COVID

Long COVID (LC) or post-acute sequelae of SARS-CoV-2 infection (PASC) refers to a variety of symptoms that persist for weeks or months after the acute phase of a SARS-CoV-2 infection has resolved [278,279]. LC can affect individuals despite the severity of the acute infection, significantly impacting their quality of life [280]. The symptoms of LC can vary widely and may include fatigue, shortness of breath, cognitive impairment, chest pain, headaches, muscle and joint pain, insomnia, mood changes, loss of smell or taste, and heart palpitations [281]. In this scenario, the increased expression of PRRs (including TLR4, cGAS and STING) highlights their crucial role in the development and maintenance of the long-term effects of SARS-CoV-2 (Table 5). Indeed, patients who experienced long-term post-COVID-19 symptoms had higher levels of cGAS, STING and IFN-α than individuals who did not experience LC symptoms [236]. Elevated levels of cGAS and STING perpetuate a systemic inflammatory state, which could lead to thromboembolic changes and multiple organ failure [232,282,283]. Cognitive impairment is another physical disorder reported in LC [284,285,286]. It has been demonstrated that infusing S protein into the mouse brain causes delayed cognitive deficits but the early TLR4 inhibition effectively prevents impairments to synapses and memory [287]. Furthermore, patients with mild SARS-CoV-2 infections who had the GG genotype of the TLR4-2604G>A variant exhibited increased TLR4 expression and were at a higher risk of cognitive impairment than those with the GA genotype. These results emphasize the important role of TLR4 in causing cognitive problems in humans and rodents and highlight its potential as a therapeutic target [287]. In fact, astrocytes, oligodendrocytes, endothelial cells and neurons also express TLRs, thereby contributing to neuroinflammation. This is supported by the detection of elevated levels of TLR2 and TLR4 in the brains of people who died from complications resulting from SARS-CoV-2 infection [288]. Furthermore, variations in the expression of MDA5 and OAS2 have been observed in children and adolescents with LC, compared with recovered patients without LC symptoms (matched controls, MC) and healthy controls. Age appears to play a key role in shaping the immune response to LC, with clear differences in IFN-related gene expression between children and adolescents [279]. In fact, although children typically experience mild COVID-19, some develop long-term symptoms months later [289]. These observations underscore the complex interplay between innate immune sensors and the persistence of long COVID symptoms. Sustained activation of PRRs may contribute to systemic and neuroinflammatory processes, suggesting that targeting PRRs could help the management of long-term symptoms.

## 7. Conclusions

Recognition of SARS-CoV-2 by PRRs leads to the activation of the IFN-dependent innate immune response. Both membrane-bound PRRs and most cytosolic molecular sensors detect SARS-CoV-2 components and activate downstream signalling pathways. These pathways converge on NF-κB and IRF3/7, thereby driving the production of pro-inflammatory cytokines and type I/III IFNs and establishing an antiviral state. Recent studies have revealed that various PRR-mediated innate immune signals are triggered on the membranes of organelles such as the Golgi apparatus, endosomes, and mitochondria [290]. These findings suggest that the dysregulation of PRR membrane trafficking may contribute to the immunopathogenesis of SARS-CoV-2 infection [290]. Similarly to PRRs, a specific group of inhibitory receptors known as inhibitory pattern recognition receptors (iPRRs) are involved in maintaining homeostasis and immune balance [291]. iPRRs also play a role in infections, including SARS-CoV-2, where they contribute to the modulation of the inflammatory response [292]. 

It is widely acknowledged that the ability of SARS-CoV-2 to evade the host immune sensors significantly contributes to delayed or insufficient activation of the IFN response. This facilitates efficient viral replication and COVID-19 progression [293]. Conversely, SARS-CoV-2 modulates these cellular pathways in order to create a favourable environment for itself. This results in the abnormal activation of late-stage components of innate immunity and, consequently, harmful IFN production [294]. Notably, the activation of inflammatory pathways, such as the NLRP3 inflammasome and the cGAS-STING axis, can lead to the excessive release of cytokines [295], contributing to the cytokine storm that is a hallmark of severe cases of SARS-CoV-2 infection [237,282,296,297,298,299]. 

A better understanding of how SARS-CoV-2 interferes with innate immune pathways could inform the development of new antiviral strategies, such as PRR agonists and antagonists. Similarly, a deeper understanding of the molecular interactions between SARS-CoV-2 and host PRRs could improve the design of vaccines, elicit stronger and broader immune responses and providing better protection against existing and emerging SARS-CoV-2 variants. 

It is notable that the engagement of PRRs by viral components, such as those from SARS-CoV-2, can induce a sustained, heightened state of innate immunity known as ‘trained immunity’, thereby enhancing resistance to secondary infections. This suggests that vaccines could be developed to harness trained immunity by incorporating suitable PRR ligands [300]. 

Since PRRs play a key role in the persistence of the long-term effects of SARS-CoV-2 infection, clarifying the underlying mechanisms is also essential to improving patient management. Potential publication bias and study heterogeneity must be acknowledged, particularly since the available data originates from specific geographical and socioeconomic contexts. Nevertheless, therapeutic strategies remain relevant in both high- and low-resource settings. Furthermore, there are significant knowledge gaps in our understanding of PRR-mediated responses during SARS-CoV-2 infection and its long-term effects. 

This review outlines future perspectives that could help to address these gaps and guide further research in this area (Table 6). The universal therapeutic gap, arising from the lack of targeted interventions against SARS-CoV-2 immune evasion, highlights the urgent need for effective, accessible, and scalable treatments, especially in low-resource contexts, where limited access can exacerbate the disease’s burden and long-term complications. International, multicenter collaboration is therefore crucial in supporting the development and validation of such therapies. Ultimately, strategies that promote equity would strengthen global prevention efforts and enhance the effectiveness of interventions against current and future SARS-CoV-2 variants, as well as potentially new pandemic viruses.

## Figures and Tables

**Table 5:** Effect of PRRs activation in long COVID.

PRR	Effect	Reference
cGAS-STING	Elevated levels lead to the onset of low-grade inflammatory diseases, including cardiomyopathy	[236]
		
TLR4	Increased expression is associated with cognitive deficits and synapse loss	[287]
		
TLR2 and TLR4	Elevated levels founded in the brains of died COVID-19 patients	[288]
		
OAS2 and MDA5	Differential expression between healthy controls (HC), matched controls and LC cases	[279]

For each PRR listed, the effects of its modulation on long COVID manifestations are summarized. Abbreviations: cGAS-STING: cyclic GMP-AMP synthase-stimulator of interferon genes; TLR2, Toll-like receptor 2; TLR4, Toll-like receptor 4; OAS2, 2′-5′-oligoadenylate synthetase 2; MDA5, melanoma differentiation-associated protein 5.

**Table 6:** Knowledge gaps and future directions in PRR-mediated response in SARS-CoV-2 infection and long COVID.

Knowledge Gaps		Future Directions
Viral evasion mechanisms of SARS-CoV-2 and their impact on PRR and innate immune response in different human tissues		Development of targeted PRR agonists/antagonists to prevent severe COVID-19 and long COVID
		
Long-term effects of PRR modulation on the development of long COVID (neurological, cardiovascular and metabolic symptoms)		Longitudinal studies on PRR and IFN-I/III expression to monitor long-term post-infection outcomes
		
Impact of emerging variants on PRR functional relevance and treatment efficacy		Evaluation of immune responses and PRR modulation against emerging SARS-CoV-2 variants, including the efficacy of vaccines or combination therapies in preventing persistent long COVID symptoms
		
Influence of age-, sex- and genetics-related differences (including TLR, OAS and MDA5 polymorphisms) on PRR activation and related immune responses		Integration of genomic and transcriptomic data to personalize antiviral and immunomodulatory therapies
		
Combined effects of PRR-modulating drugs (agonists and/or antagonists) or vaccines on long COVID		Development of predictive biomarkers for long COVID based on PRRs and IFN
Limitations of current studies: geographic bias, small sample sizes, heterogeneous protocols		International multicenter studies to validate PRR and IFN findings across distinctive populations and settings.

The main knowledge gaps concerning PRR modulation in SARS-CoV-2 infection and Long COVID are reported, emphasizing areas that need more research. For each gap, potential directions for future research are suggested, including developing PRR agonists or antagonists, evaluating immune responses to emerging variants, analyzing genetic and demographic factors, and investigating targeted strategies to prevent or mitigate the long-term effects of infection.

## Data Availability

No new data were created or analyzed in this study.
